# Improving estimates of environmental change using multilevel regression models of Ellenberg indicator values

**DOI:** 10.1002/ece3.4422

**Published:** 2018-09-06

**Authors:** Tadhg Carroll, Phillipa K. Gillingham, Richard Stafford, James M. Bullock, Anita Diaz

**Affiliations:** ^1^ Department of Life and Environmental Sciences Faculty of Science and Technology Bournemouth University Poole UK; ^2^ NERC Centre for Ecology and Hydrology Wallingford UK

**Keywords:** biodiversity change, ecological indicators, hierarchical Bayes, historical plant assemblage, missing data

## Abstract

Ellenberg indicator values (EIVs) are a widely used metric in plant ecology comprising a semi‐quantitative description of species’ ecological requirements. Typically, point estimates of mean EIV scores are compared over space or time to infer differences in the environmental conditions structuring plant communities—particularly in resurvey studies where no historical environmental data are available. However, the use of point estimates as a basis for inference does not take into account variance among species EIVs within sampled plots and gives equal weighting to means calculated from plots with differing numbers of species. Traditional methods are also vulnerable to inaccurate estimates where only incomplete species lists are available.We present a set of multilevel (hierarchical) models—fitted with and without group‐level predictors (e.g., habitat type)—to improve precision and accuracy of plot mean EIV scores and to provide more reliable inference on changing environmental conditions over spatial and temporal gradients in resurvey studies. We compare multilevel model performance to GLMMs fitted to point estimates of mean EIVs. We also test the reliability of this method to improve inferences with incomplete species lists in some or all sample plots. Hierarchical modeling led to more accurate and precise estimates of plot‐level differences in mean EIV scores between time‐periods, particularly for datasets with incomplete records of species occurrence. Furthermore, hierarchical models revealed directional environmental change within ecological habitat types, which less precise estimates from GLMMs of raw mean EIVs were inadequate to detect. The ability to compute separate residual variance and adjusted *R* ^2^ parameters for plot mean EIVs and temporal differences in plot mean EIVs in multilevel models also allowed us to uncover a prominent role of hydrological differences as a driver of community compositional change in our case study, which traditional use of EIVs would fail to reveal. Assessing environmental change underlying ecological communities is a vital issue in the face of accelerating anthropogenic change. We have demonstrated that multilevel modeling of EIVs allows for a nuanced estimation of such from plant assemblage data changes at local scales and beyond, leading to a better understanding of temporal dynamics of ecosystems. Further, the ability of these methods to perform well with missing data should increase the total set of historical data which can be used to this end.

## 1 INTRODUCTION

Resurvey studies, where communities are resampled after years or decades have elapsed, are becoming increasingly common in ecology due to interest in how ecosystems are responding to global environmental change (e.g., Diaz, Keith, Bullock, Hooftman, & Newton, 2013; Krause, Culmsee, Wesche, & Leuschner, 2015). However, contemporaneous environmental data alongside historical data on species records are often lacking, which can hamper attempts to identify drivers of community change. As one solution, Ellenberg Indicator Values (EIVs) are widely used to infer environmental change over time where no data are available for abiotic conditions (Häring, Reger, Ewald, Hothorn, & Schröder, 2014; Krause et al., 2015; McGovern, Evans, Dennis, Walmsley, & McDonald, 2011; Newton et al., 2012; Prach, 1993; Wesche, Krause, Culmsee, & Leuschner, 2012). EIVs score plant species on an ordinal scale based on estimated optimal environmental conditions for moisture, light, soil nutrient levels, reaction (pH), and salt tolerance (F, L, N, R, and S respectively) (Ellenberg, 1988; Hill, Preston, & Roy, 2004). Typically, ecologists compare mean EIV scores of plants sampled from stands of vegetation to infer differences in abiotic conditions (Diekmann, 2003). However, use of point estimate plot mean EIVs fails to account for variation in EIV scores of plant species within sample plots, which we hypothesize could improve accuracy of inferences if included. Furthermore, incomplete species occurrence records for some or all plots may lead to inaccurate estimates of plot means and thus poor inference of environmental changes over time.

The population parameter one attempts to estimate when calculating a mean EIV score from plant occurrence records—for example, describing soil reaction (EIV R)—is the mean EIV score for all plant species able to establish at this plot given the soil pH, all other things being equal (Dupré, 2000; Ellenberg, 1988). However, as well as environmental filtering for pH, a myriad of factors, including abiotic conditions and interactions with other species present in the community, will affect the probability of a particular species establishing a local population (Grime, 1977; Keddy, 1992; Vellend, 2016). This complex filtering process leads to the diverse plant assemblages we see in nature, which in turn lead to variation in EIV scores of species within and between plots.

**Figure 1:**
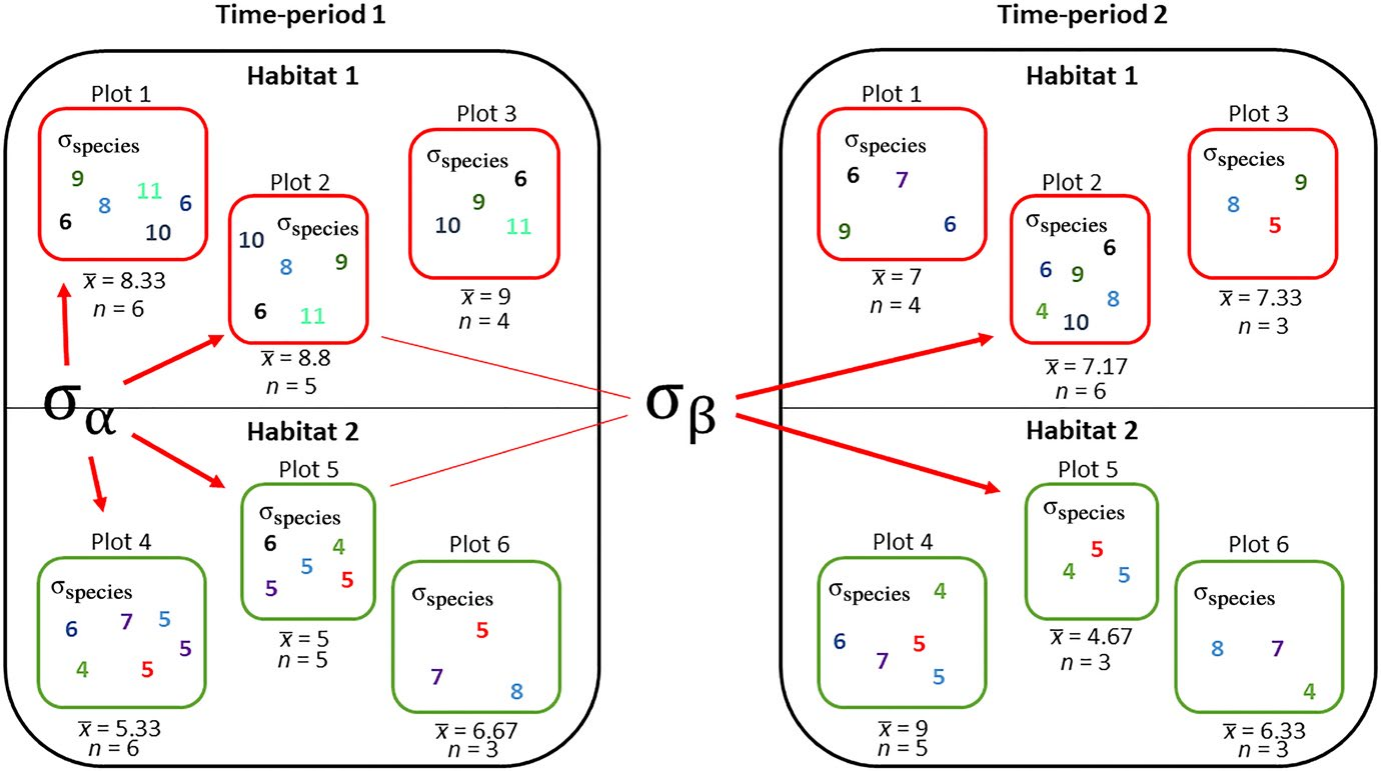
Typical spatiotemporal sampling structure of a resurvey study where Ellenberg Indicator Values (EIVs) are used to infer environmental differences underlying plant assemblages. Each color/number combination represents the EIV score of a specific plant species. In this example, plots are sampled within two separate ecological habitat types, and plant species occurrences are recorded for all plots in two separate time‐periods. The *σ* values denote components of variation (a) in EIVs among species within sampled plots (*σ*_species_), (b) in mean EIVs between plots (*σ*_*α*_), and (c) in differences in plot mean EIVs between time‐periods (*σ*_*β*_). Methods using preaveraged mean values ($\overset{¯}{x}$'s) as a starting point for inference fail to account for *σ*_species_, and as a result can lead to less reliable plot mean estimates and inferences across the wider landscape and between time‐periods

Environmental heterogeneity is an important factor in plant ecology studies generally (e.g., Maslov, 1989), and by failing to account for different levels of variation within a system, traditional methods discard much information, which may result in over‐ or underestimation of the extent of change over time (Gelman & Hill, 2006). Figure 1 depicts three distinct levels of variation that can be identified within a typical ecological study estimating environmental change using EIVs: (a) variation among EIV scores of species recorded within sampled plots (*σ*_species_); (b) variation between plots in mean EIV scores (*σ*_*α*_); and (c) variation in between time‐period differences in plot mean EIVs as environmental conditions change differentially across a landscape over time (*σ*_*β*_). Traditional methods using point estimates of mean EIVs from sampled plots (the $\overset{¯}{x}$'s in Figure 1) to infer differences between groups of plots in space or time—either broken down by a grouping factor (e.g., habitat type), or on average across all sample plots—fail to incorporate variation within plots in species EIV scores (*σ*_species_).

The suitability of hierarchical modeling to account for structure and variability in ecological systems is well established (Cressie, Calder, Clark, Hoef, & Wikle, 2009; Kéry & Royle, 2016; Royle & Dorazio, 2008), and this approach provides an ideal framework to account fully for the structure and variability identified in Figure 1. Instead of using point estimates of mean EIVs, data enter the model as species‐specific EIV scores, and inferred plot means—with all of their associated uncertainty—are estimated and used at a higher level within the model to infer differences between groups of plots in space and time (McElreath, 2016). In this way, information is shared between plots, with mean EIV estimates augmented through partial pooling—that is, plot‐level estimates being pulled towards the overall mean to an extent dependant on the number of species a mean estimate is composed of, and the variability of estimates between plots (Gelman & Hill, 2006). More fully accounting for uncertainty in this way should lead to more reliable estimates of individual plot mean values, and of differences between groups of plots in space and time (Gelman & Hill, 2006). Furthermore, because estimates are pooled according to shared information content, differences between any pair or combination of individual plots or habitats in the system can be inferred without having to contend with the issue of multiple comparisons, which should provide more power to detect change over time in conventional null hypothesis testing frameworks (Gelman, Hill, & Yajima, 2012).

A multilevel (hierarchical) modeling approach may also help to improve estimates of plot mean EIVs in instances where lists of recorded species are incomplete for some or all plots. Incomplete sampling is a common nuisance in ecological studies as some species are more difficult to detect than others, and ease of detection may vary depending on the time of year a particular plot is sampled, and among species (Chen, Kéry, Plattner, Ma, & Gardner, 2013; Kéry, 2004; Kéry & Gregg, 2003). This issue may be further compounded if recorders with differing botanical skills sample different plots, or in resurvey studies where it can be difficult to confirm the completeness of records, and where differing sampling methods may have been used. As long as data are not missing systematically across all plots, multilevel modeling should improve mean estimates for plots with missing data—and any inference based on these estimates—by pooling information across plots.

The aim of this study was to demonstrate how hierarchical modeling can lead to higher discriminatory power than traditional methods when using EIVs to assess environmental changes underlying plant communities. This is achieved by accounting for uncertainty at all levels of the ecological system and by explicitly identifying and estimating components of temporal and spatial variation in plot mean EIVs.

We demonstrate the utility of this method in studies with both complete and incomplete plot records for species occurrence by fitting models to a real resurvey dataset. The models describe two scenarios: (a) A set of plots across a landscape, resampled in a second time‐period, assumed to be replicates of a similar habitat type; and (b) a similar set of plots sampled in two time‐periods, but in this case groups of plots differ by some grouping factor (e.g., habitat type in our case study). We ask: (a) Do inferences on changes in environmental conditions in space and between time‐periods differ between hierarchical models of EIVs with a full multilevel structure and models using point estimates of raw mean EIVs from sampled plots as data, to an extent that will effect conclusions about change in the system? (b) Do hierarchical models improve mean estimates—and consequently inferences on temporal differences based on these estimates—for datasets where the full cohort of species is not recorded in all sampled plots? We provide code in the Supporting Information Data S1 to fit the models in R and Jags.

## 2 METHODS

### 2.1 Data

All models were fitted to a real ecological dataset for EIVs describing moisture, light, soil nutrient levels, reaction (pH), and salt tolerance (F, L, N, R, and S, respectively) from the PLANTATT dataset which provides EIVs adjusted for use in the UK and Ireland (Hill et al., 2004). Historical data were collected by Cyril Diver and contemporaries in the 1930s from the Studland Peninsula, Dorset, UK (Lat: 50.66, Lon: −1.9) (Diver, 1938; Good, 1935). The Peninsula consists of a habitat mosaic (~3 km^2^) characterised as dune, dune heath, tertiary heath, woodland, harbour shore, marsh, and edge aquatic plant assemblages. Diver and colleagues recorded lists of species occurrences in 74 sample plots (“compartments”) which varied in size and shape (size in m^2^: min = 899.98, max = 200764.4, mean = 44452.52), and were based on the topographical properties and local ecological characteristics of Studland (Diver, 1938). The sampling compartments of Studland fall somewhere between Permanent and Quasi‐permanent categories by the framework presented in Kapfer et al., 2017, as though they were relocated using various physical indicators and detailed ordinance survey maps, the precise boundaries between them may not always be in the exact same positions for historical and contemporary sampling. The National Trust resurveyed the area between 2013 and 2015 in a citizen science initiative coined “The Cyril Diver Project” following Divers’ original sampling plots. (https://www.nationaltrust.org.uk/studland-beach/features/the-cyril-diver-project). Both sampling and resampling efforts aimed to record all species present in their respective time‐periods by repeatedly visiting plots throughout the year and scouring them carefully in teams for the duration of respective study periods. The number of species in each sample in each plot time‐period, area, and coordinates of sample plots is detailed in Supporting Information Data S2.

### 2.2 Models

#### 2.2.1 Estimating environmental change over time in resurvey studies

The first scenario we consider is one in which we estimate between time‐period differences in mean EIVs for a resurvey study, where sample plots are considered replicates of similar, homogenous stands of vegetation in the same type of habitat. As such, model M1 below is equivalent to compiling a series of t‐tests, one for each plot, to estimate differences in mean EIV scores at plot level—although to use it for statistical testing in this manner would require major corrections for multiple testing. We formulate this simple linear model to emphasize fully the progression from fixed effects models with no‐pooling, to those with partial pooling under a multilevel structure—and to use as a baseline against which to compare plot mean estimates from hierarchical models. The appropriateness of using mean values of ordinal EIVs and means of ordinal values more generally has been widely discussed in the literature and is not the topic of this paper; however, we agree that it has proven a useful method in applied plant ecology and should continue to be so (Diekmann, 2003; Pasta, 2009).

$$\begin{array}{c} \text{M1}\\ y_{i}∼N\left(\alpha_{j[i]}+\beta_{j[i]}x_{i},\sigma_{\mathrm{species}}\right),\;\text{for}\;i=1,\ldots ,n \end{array}$$

*y*_*i*_ is the EIV score for species *i* in plot *j*, and *σ*_species_ is the estimated residual variance for EIV scores of *n* species within sampled plots. In this no‐pooling model, the *α*_*j*_ values are the plot means from time‐period 1, and each *β*_*j*_ parameter is an estimate of the difference in mean EIV in compartment *j* between time‐periods 1 and 2. *x*_*i*_ is the binary (0,1) predictor for the time‐period that species *y*_*i*_ was sampled in.

To move from “no‐pooling” to hierarchical models, we allow the *α*_*j*_'s and *β*_*j*_'s from model M1 to share information through partial pooling, changing them from fixed to random effects. As such, model M2 below can be viewed as a type of mixed effects model which allows us to use more conservative estimates of plot‐level between time‐period differences (slopes) by sharing information content across plots and thus arrive at a more accurate estimate of overall change.

$$\begin{array}{c} \text{M2}\\ y_{i}∼N\left(\alpha_{j[i]}+\beta_{j[i]}x_{i},\sigma_{\mathrm{species}}\right),\;\text{for}\;i=1,\ldots ,n\\ \left(\alpha_{j},\beta_{j}\right)∼\text{MVN}\left((\mu_{\alpha },\mu_{\beta }\right),\left(\sigma_{\alpha },\sigma_{\beta },\rho \sigma_{\alpha }^{2}\sigma_{\beta }^{2}\right)),\;\text{for}\;j=1,\ldots ,j \end{array}$$

Slope and intercept parameters are constrained to come from bivariate normal distribution (MVN) with mean vector $\left(\mu_{\alpha },\;\mu_{\beta }\right)$ to account for correlation between them (Gelman & Hill, 2006). The covariance matrix is defined by the variance in plot intercepts $\left(\sigma_{\alpha }\right)$ and slopes $\left(\sigma_{\beta }\right)$, and the covariance between the two sets of parameters $\left(\rho \sigma_{\alpha }^{2}\sigma_{\beta }^{2}\right)$, where ρ is the correlation coefficient. Allowing information on temporal differences across plots to be shared in this way makes sense particularly if the sampled plots come from a spatial area within which we expect abiotic drivers of change to be linked.

#### 2.2.2 Inferences between plots differing by a grouping factor

Sampled plots may differ by some categorical factor (e.g., Habitat type, grazing regime, etc.). We can extend model M2 to include a group‐level predictor within the sub‐models of *α*_*j*_’*s* and *β*_*j*_'s. Thus plot‐level estimates in model M3 below are improved when groups of plot differ by habitat, as now the estimates are pooled toward the habitat‐level mean value rather than the mean across all plots. M3 also allows us to estimate differences in mean changes at habitat level.

$$\begin{array}{c} \text{M3}\\ y_{i}∼N\left(\alpha_{j[i]}+\beta_{j[i]}x_{i},\sigma_{\mathrm{species}}\right),\;\text{for}\;i=1,\ldots ,n\\ \left(\alpha_{j},\beta_{j}\right)∼\text{MVN}\left(\mu_{\alpha [k]},\mu_{\beta [k]}\right),\left(\sigma_{\alpha },\sigma_{\beta },\rho \sigma_{\alpha }^{2}\sigma_{\beta }^{2}\right)),\;\text{for}\;j=1,\ldots ,j \end{array}$$

In hierarchical model M3, the data (*y*_*i*_) still enter the model at the level of plant species within plots, and the plot intercepts and slopes are still constrained to come from a multivariate normal distribution. Here, however, the means of this distribution $\left(\mu_{\alpha [k]}\right)$ and $\left(\mu_{\beta [k]}\right)$ take on a different value for each of *k* groups (habitat types in our case study). *σ*_*α*_ and *σ*_*β*_ now estimate variation in plot‐level intercepts and slopes respectively, after taking habitat type into account.

Model M3 allows us to estimate differences between groups of plots by essentially nesting a two‐way ANOVA within the model structure. To compare inferences on habitat‐level differences from the hierarchical model with those using point estimates of mean EIVs as data, we fitted generalized linear mixed models (GLMMs) with plot ID as a random effect nested in time‐period to account for repeat sampling. While this technically is a hierarchical model, it does not incorporate the multilevel structure which is the focus of this study. We compared these models to their hierarchical (multilevel) counterparts in terms of differences in magnitude, precision, and sign of habitat level estimates, and whether differences in habitat‐level EIVs between time‐periods were significant at the standard *α *= 0.05 significance level. To perform these tests of significance, habitat‐level differences in EIVs for each GLMM were corrected for multiple comparisons using the multcomp package in R (Hothorn, Bretz, & Westfall, 2008). We also calculated Bayesian *R* ^2^ for each level within the hierarchical models (data level, varying intercepts, and varying slopes) (Gelman & Pardoe, 2006).

#### 2.2.3 Analyses with incomplete species records

We refitted the models with incomplete sets of species artificially subsampled from a selection of plots to test model performances in predicting plot mean EIVs where not all species present in a plot are recorded. As improving plot mean EIV estimates by pooling information across plots—and thus improving inferences based on these estimates—is the mechanism by which we suggest that multilevel modeling is an improvement on methods using point estimates of plot mean values as data, this missing species analysis also served as our most important validation procedure (following Lin, Gelman, Price, & Krantz, 1999). If these methods can accurately estimate plot mean values primarily from information shared across plots, with most of the species missing from the focal plot, then it is clear that the models use the pooled information in a valuable way.

Plots were chosen in a random stratified manner; one plot with >50 recorded species from each habitat type in each time‐period (14 total). About 90% of species in each of these 14 plots were selected at random and excluded from the dataset, representing severe undersampling. Models M1, M2, and M3 were refitted and model outputs compared to the raw mean values when all data were included, under the assumption that plots with >50 species provided an adequate estimate of the “true mean” value. This process was repeated iteratively 120 times with a different random 90% of species removed from each plot during each iteration. Model performances were compared graphically, and using calculated summary statistics to assess precision and accuracy of plot level estimates for plots with missing species. Precision was assessed as the mean width of 50% and 95% credible intervals of plot estimates, and as the inverse variance of plot mean estimates. Accuracy was assessed as the proportion of times the “true mean” value was within the 50% and 95% credible intervals, and as the mean distance of point mean estimates from the “true mean” value.

### 2.3 Software and validation

Models were fitted in JAGS and R version 3.3.1 using package runjags with minimally informative priors following Gelman & Hill, 2006 (see Supporting Information Data S1 for a description of the models in the Jags language) (Denwood, 2016; R Core Team 2017). Additional R packages were used for analyses of mcmc chains and graphics (Plummer, Best, Cowles, & Vines, 2006; Wickham, 2009). In addition to the validation discussed in Section 2.2.3, we performed a range of posterior predictive checks and comparisons between simulated and real‐world datasets to assess model adequacy (following Gelman & Hill, 2006; Kéry & Schaub, 2012).

## 3 RESULTS

### 3.1 Analyses with incomplete species records

**Figure 2:**
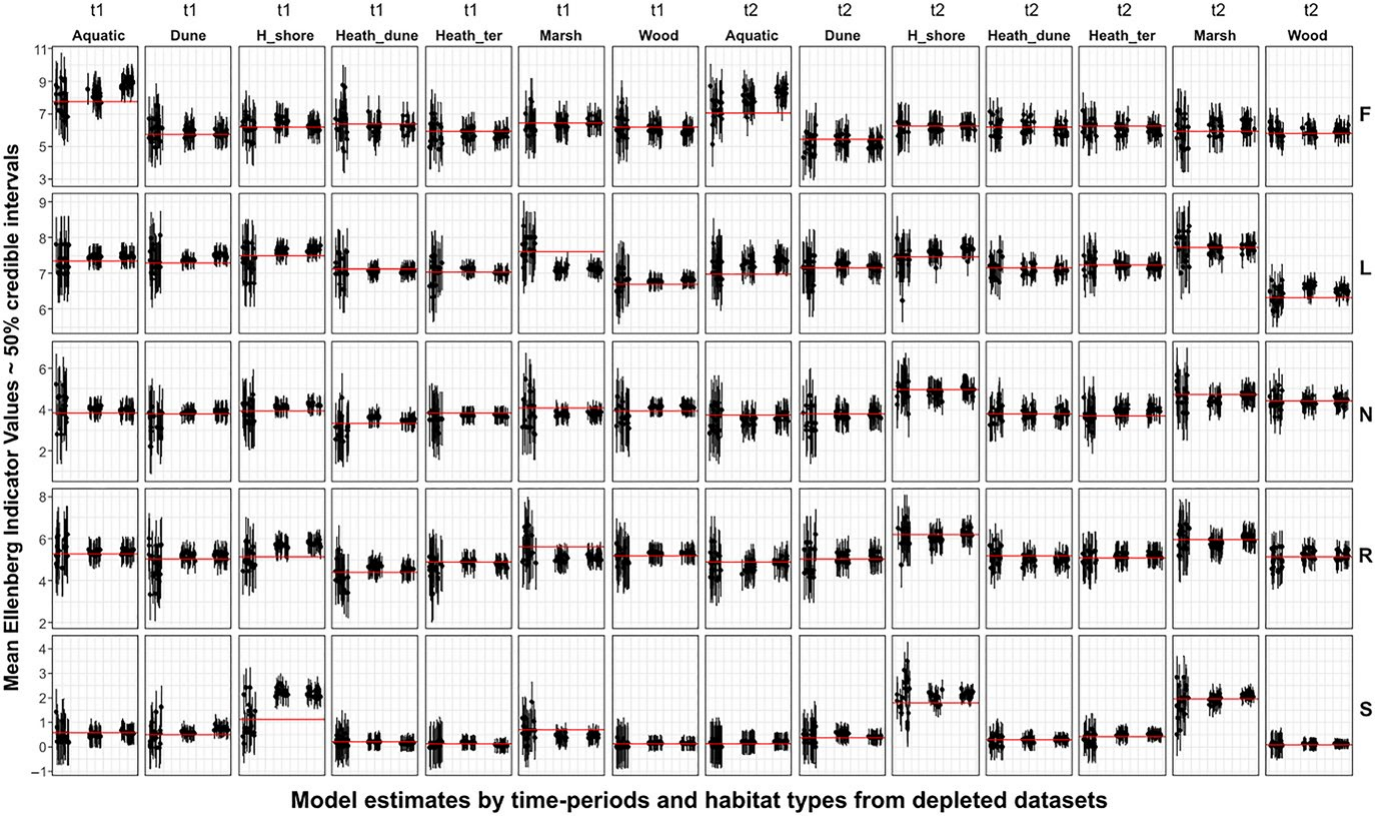
Mean estimates with 50% uncertainty intervals of plot‐level Ellenberg Indicator Values (EIVs) F, L, N R, and S from plots with a random 90% of species removed. One plot with 50 or more recorded species was randomly chosen from each habitat type in each of two sampling periods. Red lines are plot mean EIVs with full cohort of species remaining. The three clouds of points from left to right in each grid panel display uncertainty intervals from: (a) No‐pooling models, representing raw mean estimates of 10% of species randomly remaining in each iteration; (b) Estimates from hierarchical models with partial pooling of plot intercept and slope parameters, and; (c) Estimates from hierarchical models with partial pooling including group level habitat predictors. Plot shows a subset of 20 of 120 iterations run in total for clarity

Multilevel model estimates from both models M2 and M3 were consistent across separate runs of the simulation, regardless of which 10% species remained, with high levels of precision and accuracy (Figure 2, Table 1). Plot mean estimates with missing species were closer to the true means for hierarchical vs. nonhierarchical models for all four EIVs, often by more than a factor of two—averaging across replications and depleted plots (Table 1). The proportions of “hits” for 50% and 95% credible intervals about the mean estimates differed between models and EIVs, but underperformed for some hierarchical models due to consistent misses across replications for some individual sample plots (Figure 2). Models without group‐level habitat predictors performed slightly better in this respect (Table 1).

**Table 1:** Model performances from analyses of plots with a random 90% of species removed. All statistics were calculated for 14 depleted plots over 130 simulations of the validation analysis

Model	Mean width 0% CI	Mean width 95% CI	Mean precision	Proportion of hits 50% CI	Proportion of hits 95% CI	Avg. dist. from true mean
EIV F						
RV M1	0.87	2.54	3.97	0.53	0.96	0.51
RV M2	0.61	1.77	14.58	0.58	0.96	0.32
RV M3	0.51	1.5	27.48	0.53	0.87	0.36
EIV L						
RV M1	0.46	1.33	11.52	0.52	0.96	0.25
RV M2	0.2	0.6	412.84	0.46	0.89	0.15
RV M3	0.19	0.57	429.94	0.39	0.82	0.17
EIV N						
RV M1	0.83	2.41	4.02	0.54	0.97	0.46
RV M2	0.36	1.07	193.62	0.45	0.99	0.2
RV M3	0.39	1.05	170.69	0.58	1	0.16
EIV R						
RV M1	0.78	2.25	4.45	0.5	0.97	0.44
RV M2	0.39	1.16	77.89	0.5	0.92	0.26
RV M3	0.37	1.09	96.02	0.55	0.9	0.21
EIV S						
RV M1	0.54	1.58	32.99	0.64	0.92	0.3
RV M2	0.24	0.72	671.08	0.66	0.89	0.18
RV M3	0.21	0.62	605.52	0.55	0.85	0.19

### 3.2 Plot‐level inference

**Figure 3:**
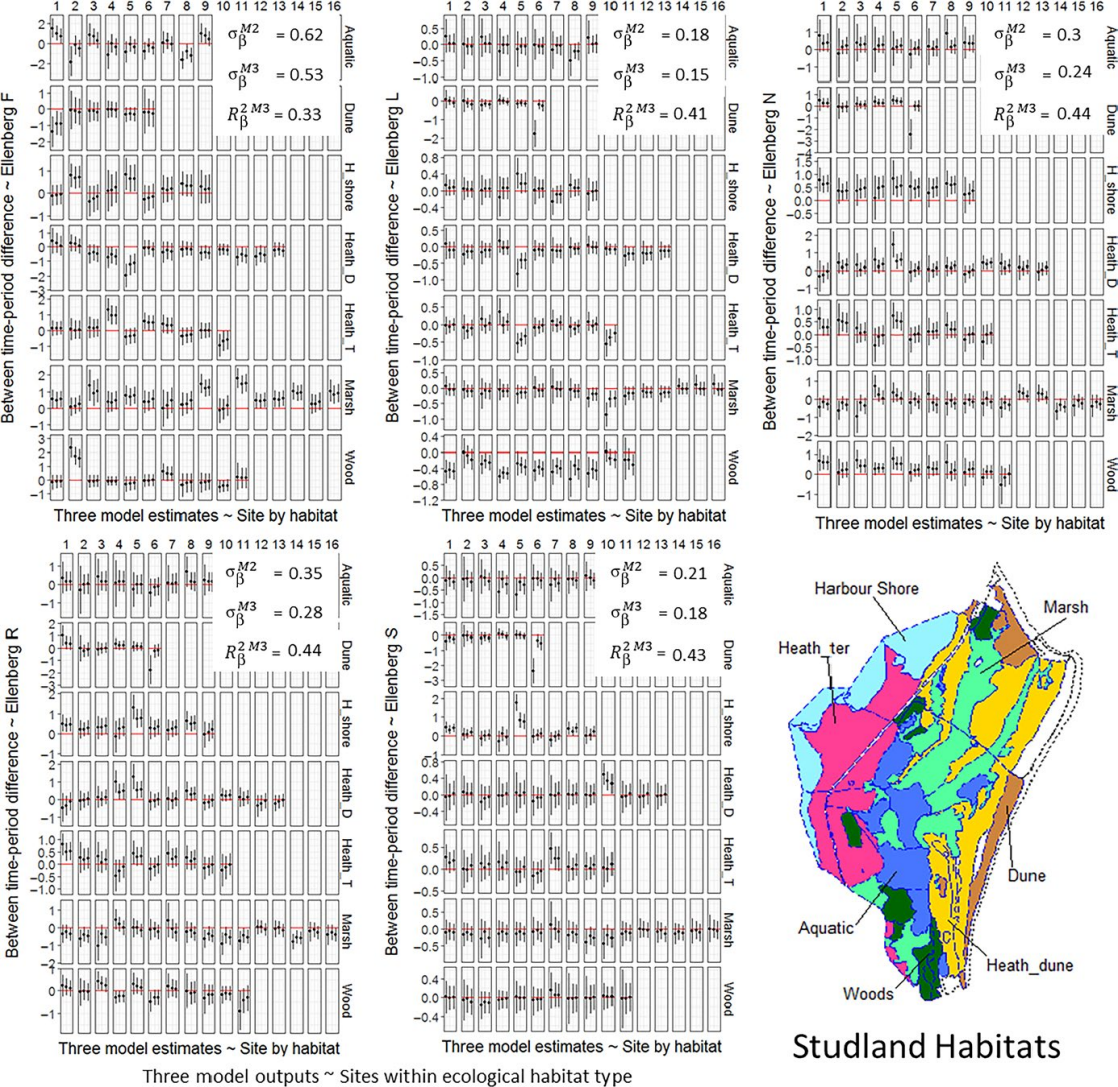
Changes in Ellenberg Indicator Values (EIVs) between sampling in the 1930s and resampling in 2010s. Plots show mean and 95% Bayesian credible intervals for estimates of plot‐level changes between sampling periods for each of seven habitats (Map inset). Each grid cell contains varying slope parameters (*β*'s) from models M1 (no‐pooling), M2 (hierarchical), and M3 (hierarchical) from left to right, respectively. Horizontal red lines indicate zero change between sampling periods. White textboxes show unexplained variance in slope parameters in hierarchical models with and without habitat as a predictor ($\sigma_{\beta }^{M2}$ and $\sigma_{\beta }^{M3}$, respectively) and the estimated proportion of variance explained by habitat as a group‐level predictor for slope parameters (Bayesian *R* ^2^). Each column represents numbered plots within habitat types

Out of sample predictive accuracy was markedly better in hierarchical vs. nonhierarchical models for all five EIVs as estimated by DIC (ΔDIC between 8.6 and 40), and models including group‐level habitat predictors (M3) were invariably the best by this criteria (Table 2). Estimates of variance among species EIVs within sample plots (*σ*_species_) from hierarchical models were much larger in all cases than between plot (*σ*_*α*_) and between time‐period (*σ*_*β*_) variance estimates. The inclusion of ecological habitat type in the M3 models significantly reduced residual variance in plot‐level intercepts and slopes (*σ*_*α*_ and *σ*_*β*_) for models of all EIVs. The extent to which intercepts and slopes were pooled $\left(\lambda_{\alpha }\right)$ and $\left(\lambda_{\beta }\right)$ differed between models of the five EIVs, but was much higher for model M3 versus M2 in all cases, which exemplifies how adding habitat type provided a better target for pooled estimates by reducing residual variance in plot‐level parameter estimates (Table 2). The inclusion of ecological habitat type in the M3 models explained over 40% of variation in the pooled plot‐level slope parameters for EIVs L, N, R, and S, while it explained 33% for EIV F, which also had higher estimates of *σ*_*β*_ both before and after the inclusion of habitat than the other EIVs (Figure 3).

**Table 2:** Residual variance (*σ*), Bayesian *R* ^2^, mean pooling of estimates (λ), effective number of parameters (pD), and DIC values for models fit to Ellenberg Indicator Values F, L, N, R, and S of plant species from a re‐visitation study on the Studland peninsula between the 1930s and 2010s. NP are “no‐pooling,” H are “hierarchical,” and HG are “Hierarchical with group‐level predictor” models. Parameters with subscripts *α* and *β* were estimated at the level of varying intercepts and slopes, respectively

Model	*σ*_plant_	*σ*_*α*_	*σ*_*β*_	$R_{\mathrm{plant}}^{2}$	$R_{\alpha }^{2}$	$R_{\beta }^{2}$	λ_*α*_	λ_*β*_	pD	DIC
EIV F										
M1(NP)	1.74	—	—	0.22	—	—	—	—	149.1	36,391.8
M2(H)	1.74	1.09	0.62	0.22	0	0	0.05	0.25	127.5	36,383.2
M3(HG)	1.74	0.47	0.53	0.22	0.83	0.33	0.3	0.38	117.8	36,371.2
EIV L										
M1(NP)	0.91	—	—	0.12	—	—	—	—	148.9	24,561.4
M2(H)	0.91	0.28	0.18	0.12	0	0	0.13	0.41	98.7	24,526.8
M3(HG)	0.91	0.18	0.15	0.12	0.63	0.41	0.36	0.61	94.7	24,521.9
EIV N										
M1(NP)	1.64	—	—	0.06	—	—	—	—	148.8	35,330.1
M2(H)	1.64	0.33	0.3	0.06	0	0	0.24	0.42	90.4	35,290.8
M3(HG)	1.64	0.25	0.24	0.06	0.42	0.44	0.43	0.62	87.1	35,289.5
EIV R										
M1(NP)	1.56	—	—	0.08	—	—	—	—	148.9	34,100.7
M2(H)	1.54	0.45	0.35	0.08	0	0	0.17	0.42	107.5	34,071.4
M3(HG)	1.54	0.34	0.28	0.08	0.43	0.44	0.33	0.6	100.9	34,068.6
EIV S										
M1(NP)	1.08	—	—	0.22	—	—	—	—	148.9	27,596.9
M2(H)	1.08	0.56	0.21	0.22	0	0	0.06	0.52	108	27,577
M3(HG)	1.08	0.27	0.18	0.19	0.76	0.43	0.29	0.62	95	27,563.6

### 3.3 Habitat‐level inference

**Figure 4:**
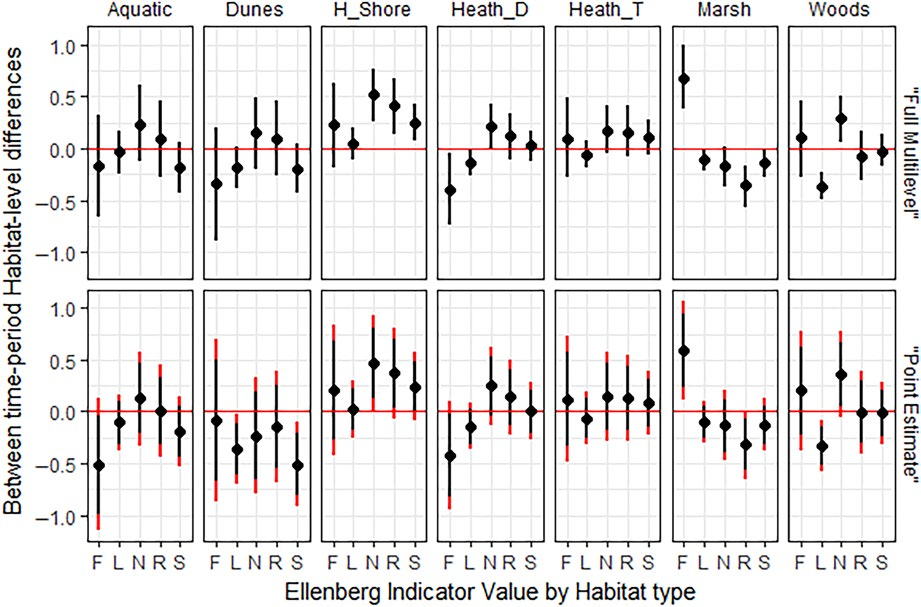
Mean and 95% Bayesian credible intervals (top) and confidence intervals (bottom) for habitat level differences in mean Ellenberg Indicator Values (EIVs) for Moisture (F), Light (L), Nutrients (N) Reaction (R) and Salinity (S) on the Studland peninsula between the 1930s and 2010s. Top row shows estimates from multilevel models with recorded species EIVs as data (model M3 from text), while the bottom row shows estimates from mixed effects models using raw means of plot EIVs as data. Red extensions to the GLMM confidence intervals represent corrections for multiple testing; hierarchical estimates do not need to be corrected due to pooling of estimates

Estimates of change in mean habitat‐level EIVs between time‐periods 1 and 2 differed to a large extent between full multilevel models (M3) and GLMMs using raw mean EIVs as data (Figure 4). While mean estimates of habitat level change were often similar between the two sets of models, hierarchical model estimates were more precise with narrower 95% Bayesian credible intervals than GLMM estimates. Furthermore, to infer differences at the standard *α* = 0.05 level as commonly practiced, GLMM confidence intervals need to be adjusted for multiple comparisons, whereas pooled estimates from hierarchical models do not (Gelman et al., 2012), which led to a rejection of a null hypothesis of no change in environmental conditions in six of 35 instances in this system using estimates from the full multilevel model where we would have to accept the null hypothesis of no change using the GLMM estimates (Figure 4). This may lead one to conclude that there has been no significant change in the harbour shore habitat from GLMM results for instance, whereas hierarchical model results show strong, precise directional change in soil nutrients (N), pH (R), and salinity (S) underlying these assemblages. Similarly, GLMM results would underestimate the extent of change in the marsh, woodland, and dune heath habitats compared with the more precise hierarchical estimates. However, despite the adjusted confidence intervals in the GLMMs, pooling of estimates in the multilevel models led to more conservative estimates of change in the dune habitat, which would lead us to conclude minimal change over time (accept the null hypothesis of no change) for EIVs L and S, while we would conclude stronger negative change from GLMM estimates (reject the null hypothesis of no change).

## 4 DISCUSSION

We have shown that multilevel modeling provides improved discriminatory power when estimating differences in mean Ellenberg indicator values between historical and contemporary plant assemblages, both at the level of individual plots and across the wider community. Multilevel models suggested a prominent role of hydrological changes—alongside succession processes—in driving compositional change between sampling periods in our case study, the extent of which would not be revealed by inference using point estimates of plot mean EIVs as data. When we removed 90% of plant species from a selection of species rich plots, estimates of plot mean EIVs from hierarchical models were very close in the majority of cases to mean values with the full cohort of species remaining. This was in stark contrast to raw means for randomly remaining species, and it demonstrates the rich potential for improving estimation and inference by pooling information across plots in hierarchical models in instances of nonsystematic missing data, which are common in ecological studies. Taken together these findings highlight the potential value of information discarded when point estimates of plot mean EIVs are used as the starting point for inference and show how hierarchical modeling can increase the utility of EIVs in suggesting the nature of environmental factors likely underlying changes in plant community composition.

### 4.1 Model performance with missing data

The phenomenon of recorders overlooking species present when performing surveys is a consistent feature of ecological sampling and can lead to bias in estimates of many ecological rate and state variables (Chen et al., 2013; Kéry, 2004; Kéry & Gregg, 2003). While missing species may not be an issue when using weighted averages of EIVs (Ewald, 2003), our analyses on artificially depleted plots for presence/absence data show the utility of hierarchical models to help alleviate inaccuracy in estimates due to imperfect sampling and non‐systematic missing data. The ability of the multilevel models to estimate plot mean EIVs accurately when the majority of species are missing should also allay any apprehensions over using the ordinal EIVs fit to a Gaussian distribution at the data level of these models; improvement in plot mean values is the primary aim of this study and results from analyses on depleted datasets demonstrate that this has been achieved.

### 4.2 Habitat‐level inference

Hierarchical model performances improved with habitat type as a group‐level predictor by providing better targets for pooled estimates. Furthermore, the ability to infer change over time from resulting habitat estimates without correcting for multiple comparisons allows us to build a more nuanced and precise picture of environmental change over time. Effect sizes for changes in habitat level mean EIVs in the Studland case study were small (<1) in all cases, but as these specify average changes across entire habitats they still indicate meaningful directional changes in environmental conditions. With small effect sizes—as will usually be the case given the scale on which EIVs are quantified—the increased precision of estimates gained from hierarchical modeling is a major advantage in revealing the direction and magnitude of environmental change in a study system.

Broad increases in EIV N across the habitats of Studland are in‐line with studies over a similar period both across the county of Dorset (Newton et al., 2012) and further afield (Bennie, Hill, Baxter, & Huntley, 2006), possibly resulting from atmospheric nitrogen deposition. Other trends are likely more specific to Studland, including wetter conditions across the marsh habitat, and decreases in EIV L across the woodland and dune heath habitats probably indicating ecological succession. Such location specific changes in the environment could have important effects on co‐occurring animal assemblages. For example, changes in precipitation levels can lead to shifts in vegetation structure and resulting changes in rodent community composition (Ernest, Brown, Thibault, White, & Goheen, 2008). The wetter marshes of Studland may have similarly affected local invertebrate and mammal assemblages, and we have shown that hierarchical modeling is better suited to uncover such effects when using EIVs.

While pooled habitat level estimates from multilevel models suggested more widespread change across the Studland system than did estimates from the GLMMs, they were also more conservative than the GLMM estimates in an important way, exemplified by the dune habitat. Larger changes estimated from the GLMMs in dune plots result from a large influence of one plot (dune plot number 6, Figure 3), whereas in the multilevel models, the influence of this plot was dampened by the pooling of this plot's slope (*β*) estimate. In time‐period 1, this was a newly formed dune which only seven plant species had colonized. From typical dune succession, we would expect this plot to become more shaded, more acidic, and more nutrient rich over time (Jones, Sowerby, Williams, & Jones, 2008). While the raw mean estimates do suggest that it has become more shaded and more acidic by time‐period 2, they would also suggest that it has become less nutrient rich. It seems likely that the apparent decrease in soil nutrient levels in this plot is a confounded estimate driven by the strong correlation between EIV R and N (Diekmann, 2003), at a plot where soil pH was probably a stronger driver of species recruitment in time‐period 1 (Jones et al., 2008). We would suggest that without specific ecological knowledge of a plot, in general it is a worthwhile trade‐off to underweight plot mean values as the multilevel models should do, rather than overweighting it as is probable using point estimates from plots with sparse data. This should reduce overconfidence in specific plot values, giving a more accurate estimate of change in this plot despite the few plant species present in the 1930s, while also reducing the effect of outliers on habitat‐level estimates of change (McElreath, 2016).

### 4.3 Plot‐level inference

Using hierarchical models to account explicitly for different variance components in a study system, we can build a more in‐depth picture of changes that have occurred. In the Studland case study, variance in EIV scores among plant species within sample plots (*σ*_species_) was larger in all cases than variance between plots (*σ*_*α*_) and variance in plot‐level changes between time‐periods (*σ*_*β*_) for all EIVs, which illustrates the value in pooling information between plots in this way to improve estimates of plot mean values. High variance estimates within plots reflect the fact that the environmental parameter an EIV represents tends to play just a small role in determining whether a plant species occurs in a given area, and that in any sample plot only a subset of species likely to occur despite environmental constraints will do so at a given time (Pärtel, 2014). Species may be absent from plots they could potentially occupy for various stochastic and mechanistic reasons (Callaway & Walker, 1997; Chave, 2004; Leibold et al., 2004), or they may be missed by recorders in a given sampling instance as previously discussed (Kéry & Andrew Royle, 2010). Computation of separate *R* ^2^ values for variance explained by habitat type for intercept and slope parameters is also highly valuable, as practitioners will often be interested only in the changes over time, and not the baseline differences between habitat types.

The ability to provide a plot‐specific picture of local change alongside estimates of average trends across the wider landscape should also prove valuable to those wishing to concentrate on finer details to aid management, or to use as indicators of dynamics affecting ecological communities contemporaneous with plant assemblages. For example, when we look at changes in plot mean EIVs over the 80‐year period across the Studland Peninsula (*β* parameters), we see it was far more variable for EIV F (moisture) than for the other EIVs both before and after accounting for habitat type. While some changes in this system—such as levels of shade (EIV L) across the woodland plots—may have clear ecological explanations (e.g., succession) specific to habitat types, highly variable changes in plot mean EIV F estimates suggest that changes in the hydrological profile of the peninsula is an important abiotic driver of change in community composition across habitat boundaries. With hierarchical models, we can pinpoint outliers or plots within which change does not match plots in a similar habitat because pooling allows us to view each estimated plot mean in isolation with more confidence that it is a balanced estimate (Gelman et al., 2012). Inspection of these plot values could lead one to develop new hypotheses about drivers of change—for instance spatial proximity to a body of water, or height above sea level—which can be easily incorporated back into the model once data is compiled on them to assess their influence. In this way, hierarchical models can be used in conjunction with knowledge of the details of a specific system to uncover drivers of change as part of an iterative scientific process.

### 4.4 Model extensions and flexibility

The multilevel models presented here, particularly fitted in a flexible Bayesian framework, can be extended or adapted to specific study systems in many useful ways. For instance, other grouping factors—in place of or in addition to habitat type—may be added to the submodels for intercepts and/or slopes (e.g., natural vs. semi‐natural, grazing regime, management practice). Similarly, continuous predictors could be added if they are of interest (e.g., plot elevation, plot area). One could also add predictors at the level of species within plots such as %cover or invasive vs. noninvasive species, depending on specific study aims. Informative or regularizing priors may be used, which could be particularly useful in instances of small sample sizes in terms of numbers of plots or species richness within plots (McElreath, 2016). Finally, the method could be adapted for use on any quantitative trait values which are averaged across species, which may help address issues of robustness (Aiba et al., 2013).

## 5 CONCLUSIONS

The increasing prevalence of resurvey studies in plant ecology, coupled with the importance of understanding accelerating environmental change, has led to Ellenberg indicator values becoming an important tool in the ecologists’ kit. We have demonstrated how multilevel modeling can provide a more powerful discriminatory framework when using EIVs to hypothesize the nature of environmental dynamics underlying compositional change in plant communities. These methods also perform very well in situations where some or all plots sampled do not have the full cohort of species recorded. Our contribution describes one more way hierarchical modeling, particularly applied in a flexible Bayesian framework, provides an ideal way to describe the multitude of hierarchical structures we see at all levels in biological systems, from cells to meta‐communities. Furthermore, we contest that identifying and explicitly modeling components of variation within an ecological system in this way can lead to the development of further hypotheses about environmental drivers shaping plant community functional characteristics in a way that is difficult using traditional statistical techniques, as our case study demonstrates.

## CONFLICT OF INTEREST

None declared.

## AUTHOR CONTRIBUTIONS

T.M.C. conceived the study, and compiled and analyzed the data. A.D., P.G., R.S., and J.M.B contributed conceptually during planning and implementation phases improving focus and cohesion, and provided feedback and redirection on multiple drafts.

## DATA AVAILABILITY

Data on species occurrences in both time‐periods and on sampling plots are available in the Dryad repository.

Doi: https://doi.org/10.5061/dryad.r8k26cd.
